# Data cluster analysis-based classification of overlapping nuclei in Pap smear samples

**DOI:** 10.1186/1475-925X-13-159

**Published:** 2014-12-09

**Authors:** Mustafa Guven, Caglar Cengizler

**Affiliations:** Faculty of Engineering and Architecture Department of Biomedical Engineering, Cukurova University, Balcalı, 01330 Adana, Turkey

**Keywords:** Pap smear, Nuclei, Overlapped, Clustering

## Abstract

**Background:**

The extraction of overlapping cell nuclei is a critical issue in automated diagnosis systems. Due to the similarities between overlapping and malignant nuclei, misclassification of the overlapped regions can affect the automated systems’ final decision. In this paper, we present a method for detecting overlapping cell nuclei in Pap smear samples.

**Method:**

Judgement about the presence of overlapping nuclei is performed in three steps using an unsupervised clustering approach: candidate nuclei regions are located and refined with morphological operations; key features are extracted; and candidate nuclei regions are clustered into two groups, overlapping or non-overlapping, A new combination of features containing two local minima-based and three shape-dependent features are extracted for determination of the presence or absence of overlapping. F1 score, precision, and recall values are used to evaluate the method’s classification performance.

**Results:**

In order to make evaluation, we compared the segmentation results of the proposed system with empirical contours. Experimental results indicate that applied morphological operations can locate most of the nuclei and produces accurate boundaries. Independent features significance test indicates that our feature combination is significant for overlapping nuclei. Comparisons of the classification results of a fuzzy clustering algorithm and a non-fuzzy clustering algorithm show that the fuzzy approach would be a more convenient mechanism for classification of overlapping.

**Conclusion:**

The main contribution of this study is the development of a decision mechanism for identifying overlapping nuclei to further improve the extraction process with respect to the segmentation of interregional borders, nuclei area, and radius. Experimental results showed that our unsupervised approach with proposed feature combination yields acceptable performance for detection of overlapping nuclei.

## Background

Although cervical cancer is one of the most mortal cancers in women, it is highly curable if it is diagnosed at an early stage. Pap smear test is a popular gynecological scanning test to diagnose cervical cancer. It is based on interpretation of cervical cells under microscopic examination. During manual screening of cervical cytology samples, the observer searches for morphometric changes and visual abnormalities on cells [1]. The false rate ratio may be increase in this screening due to subjective variability of different observers. Moreover, manual screening is an unreasonably time-consuming and costly process due to several types of distortions such as uneven dyeing, optical errors, artifacts, overlapping cells, mucus, blood etc. on samples. Thus, there has been a great motivation for automating Pap-test to reduce human error and to decrease the time consumption [1]. An automated Pap smear screening system should be able to delineate cells within samples to classify cervical cells.

In malignant cells, nuclei may be disproportionately enlarged and irregular both in form and outline. Thus, one of the most common features that guide the detection of an existing malignancy is an increased nucleus-to-cytoplasm ratio [2]. Hence, one of the highest priority tasks for an automated Pap smear monitoring system is the segmentation of cell nuclei. Moreover, the correct interpretation of nuclei abnormality depends on accuracy of the nuclei detection mechanism in automated systems [3].

In most Pap smear samples, some nuclei overlapping occurs, which is a factor that makes automated Pap smear monitoring systems error prone [4, 5]. Overlapping cell nuclei often appear as adjacent darker regions within Pap smear samples. The appearance of these darker regions most likely cause automated systems to interpret the whole area as a single nucleus. Overlapping nuclei in the segmented region may cause the misclassification of a nucleus as abnormal. Thus, overlapping and adjacent nuclei must be distinguished prior to any further processing [3, 6].

Many studies have sought to develop methods for accurately determining the borders of overlapping cell nuclei. For instance, Jung et al. reported an unsupervised Bayesian classification scheme for separating overlapping regions [2]. In another study, Li et al. utilized a modified gradient vector flow [7], as well as radiating gradient vector flow (RGVF) snake and k-means unsupervised clustering methods, for the accurate extraction of overlapping cytoplasm and nuclei in their study. Other methods including watershed were also proposed in the literature [8]. These previous studies show that there has been a great interest in accurately determining cell nuclei borders inside adjacent regions [9]. However, it is critical that before any further separation process takes place, each nucleus should be judged as to whether it is overlapping or not. Our study objective was to develop a fully automated elimination mechanism specializing in the classification of overlapping nuclei. Our proposed model is not a segmentation approach for determining interregional borders. Furthermore, this model may judge the region even if there are no apparent interregional nuclei borders.

We used morphological operations to determine cell nuclei borders and a clustering-based decision mechanism to examine detected objects to assess the presence of single or multiple nuclei inside a region. Using this approach, several new features are extracted to optimize the success of the clustering algorithm. We prefer to use a fuzzy c-means algorithm as a clustering method in this study, as it provides an unsupervised decision mechanism capable of distinguishing different classes of cell nuclei from their previously extracted features. One of the reasons we prefer a clustering-based algorithm is that no training or learning stages are needed in clustering-based approaches. This results in flexibility in the developed system and increases the success rates in cases where multiple samples are examined due to practical requirements.

**Figure 1:**
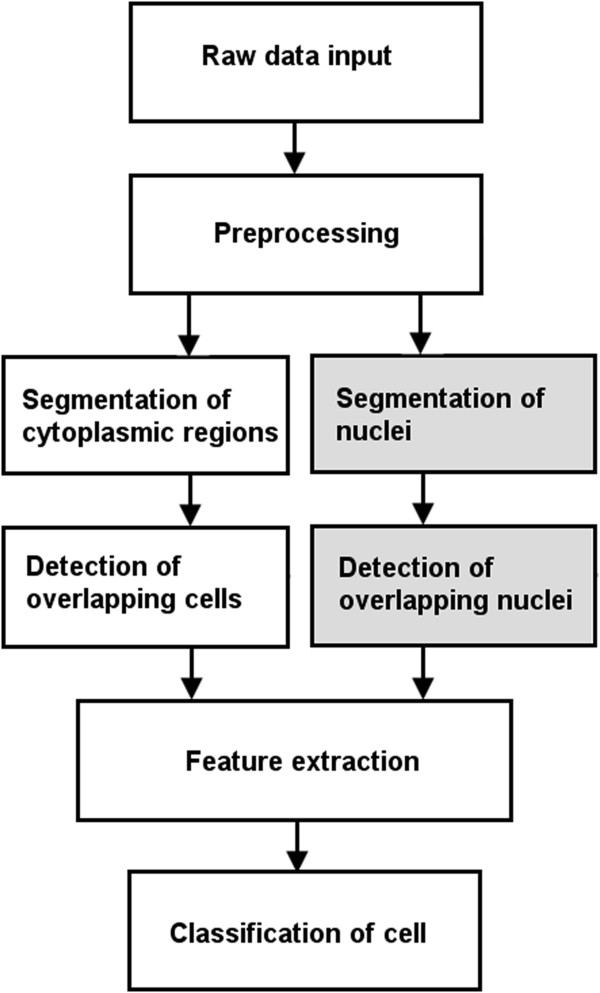
**Block diagram of a typical automated screening system.** The possible blocks of a typical automated Pap smear diagnosis system where focus range of our study is shown in the gray blocks.

In most previous studies, a typical analysis structure of an automated or semi-automated Pap smear diagnosis system includes segmentation of both the nuclei and cytoplasm regions [2, 3, 6]. According to the common structural approach, a computerized system should be able to classify and discriminate overlapping nuclei before feature extraction. A possible block diagram of such an automated system is shown in Figure 1, where the gray-filled blocks indicate the suggested position for detecting the presence of overlapping in this study. Since the major goal of our study is the development of a pre-detection mechanism for overlapping to improve the interregional border extraction process, the separation of nuclei and extraction of interregional walls will be addressed in a later study. Our aim here is to develop an unsupervised overlapping nuclei detection mechanism for automating Pap smear screening systems.

## Methods

**Figure 2:**
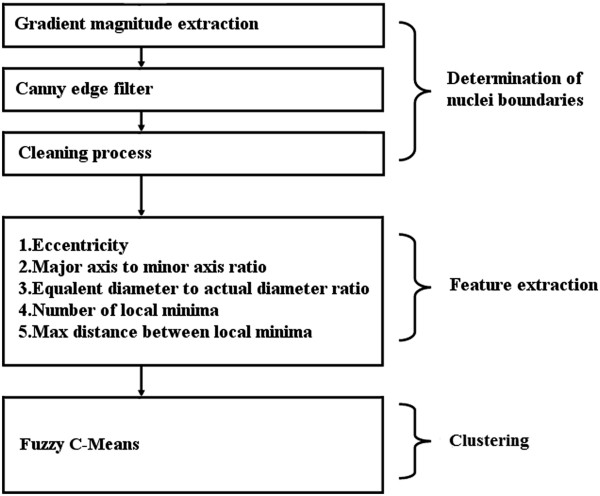
**Basic steps of our method.** Basic computational stages of the proposed approach are detailed in a block diagram.

This study contains three consecutive steps: 1) the determination of nuclei boundaries; 2) the feature extraction of cell nuclei; and 3) the determination of the presence or absence of overlapping nuclei. According to the existing algorithmic flow, candidate nuclei regions are extracted using morphological operations. Then, several features are extracted from previously segmented areas and, finally, overlapping regions are classified by clustering techniques. A block diagram of the general flow is shown in Figure 2, and the steps are described in detail in the following paragraphs. The methods we introduce here were developed using a MATLAB environment.

### A. Data set

All methods introduced here were applied to the study test set which consisted of a total of 290 nuclei within 10 cervical images where 8*%* of the detected nuclei overlapped. All images were taken from different subjects. A NIKON microscope equipped with 100× magnification is used for taking images which are processed with Papanicolaou staining. The study is performed in accordance with the Declaration of Helsinki and approved by institutional ethics committee. Ground truthing of the segmentation and classification processes was performed by two observers. While all images originally had 2560×1920 pixels, the samples were down-sized to 1280×960 pixels. All original sample images were stored in RGB color space in a JPEG format. In addition to the test set, we used a sample set of 16 public cervical cytology images from the International Symposium on Biomedical Imaging (ISBI, http://cs.adelaide.edu.au/~carneiro/isbi14_challenge/dataset.html) 14 Challenge for tuning and evaluation purpose. The set contains 690 nuclei where 14*%* of them were determined as overlapped. It should be noted that images from ISBI were not previously ranked for abnormality. Instead, we used 140 normal and 140 abnormal nuclei images from the Herlev data set (HDS) for observing the significance of the proposed feature set for abnormal and normal cell nuclei. The HDS consists of segmented single cells collected and ranked by cytotechnicians at the Department of Pathology at Herlev University Hospital and the Department of Automation at Technical University of Denmark for classification experiments. Totally 1240 nuclei including samples from Herlev data set were examined in the study.

### B. Determination of nuclei boundaries

**Figure 3:**
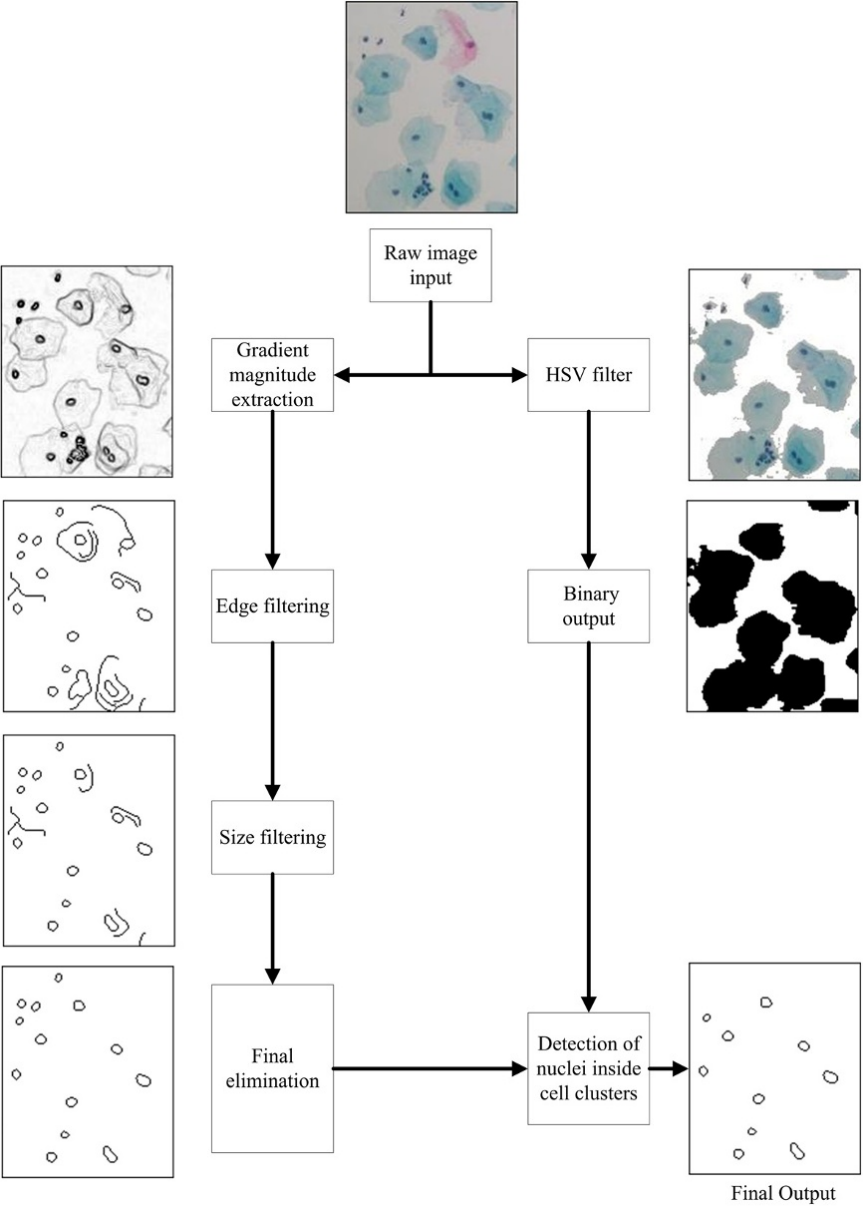
**Nuclei detection process.** All nuclei detection process is given in block diagram form with regions from sample images.

Most of the Pap smear test images in the study sample contained blood cells and artifacts. In the study, cell clusters from the test set were segmented from the background prior to the delineation process to eliminate artifacts and undesired data from outside the cytoplasmic regions. It is relatively easy to remove background pixels during the segmentation of the outer boundaries of cell clusters. Two factors that simplify this process include the color and contrast differences between the background and cluster regions. To achieve an effective and low cost extraction process, we converted our original test images from RGB to a hue-saturation-value (HSV) colormap in the range 0 and 1. Then we applied a hue filter to the images and chose the hue value limits 0.2 (lower) and 0.7 (upper). Output of the HSV filtering process is given in Figure 3. Finally, we converted the images into grayscale intensity images with 8-bit depth for further processing.

Cell nuclei appear as one of the darkest regions in most cervical samples. Other darker regions include those attributed to artifacts, mucus, blood, etc. According to global data, it is reasonable to presume that the location of the cell nuclei is in the intensity valleys [10]. Nuclei boundaries cause the formation of high gradients on images as a result of the density difference between the cytoplasm and nuclei regions [11]. Using this global information on the appearance of nuclei as a guide, we divided the nuclei extraction process into four consecutive steps in our study: 1) extraction of the gradient magnitude of the images; 2) filtering the images with an edge detection filter; 3) cleaning of some of the final images of any remaining artifacts via object size based filtering. 4) Final morphological operations for touching pixels and remaining artifacts. Sample outputs of these steps are presented in Figure 3 as a block diagram. A similar approach was used by Plissiti et al. [11], in which the authors extracted and filtered the gradient magnitude of samples to assess the initial nuclei contours.

We determined the corresponding gradient value of a sample image at a particular coordinate by combining the partial derivative of the image in the x and y directions. We converted all sample images to grayscale before beginning the work flow, and applied a Sobel operator as a discrete differentiation operator to determine the partial derivative in both directions.

In a typical gradient magnitude image of a Pap smear sample, nuclei boundaries may be much more apparent. However, final nuclei boundaries should be segmented to progress with this analysis. In the next segmentation step, the gradient of the sample image is filtered with a Canny edge detection filter to eliminate lower transition regions [11]. We determined the threshold value for the Canny edge detector to be 0.4, which is optimal for our purposes. With this threshold value, most of the samples preserved their important structural properties after the filtering process. A sample region after edge filtering process is given in Figure 3 as following stage to gradient magnitude extraction process. After the Canny edge detector filter was applied, almost all of the refined nuclei boundary data were located within the image. However, undesirable boundary data such as cytoplasm walls, artifacts, and several types of connected objects still existed within the nuclei boundaries. So, we combined a series of morphological operations to eliminate the remaining artifacts from the various data collected.

As a following stage to edge filtering, all connected objects were filtered according to their sizes to remove some of the remaining artifacts. Then, all non-connected objects were filtered. The purpose of eliminating these objects is to locate any closed-loop contours, which are likely to be nuclei boundaries. In the recent form of the output image, nuclei walls are being located as closed-contour connected pixel groups in binary images. However, in most of the cases, there may be extra pixels and pixel groups touching the nuclei walls. In the final stage, these bifurcations are removed to extract actual nuclei walls before conducting feature extraction. The scope of the filtering in this stage is shown in Figure 3 as final filtering process. Pseudo code for this elimination process is given in Algorithm 1.


In the final form of the output image, most of the nuclei boundaries were detected and filtered. However, nuclei belong to blood cells and nuclei outside the cytoplasmic regions were still within the samples. All undesired objects outside the previously detected cell clusters were removed as final step of nuclei segmentation stage. Most of the blood cells and nuclei outside the cell clusters were removed in this stage. HSV filter was eliminated most of the blood cells as a result of color and contrast differences. Final form of a sample region is given in Figure 3.

### C. Feature extraction

**Figure 4:**
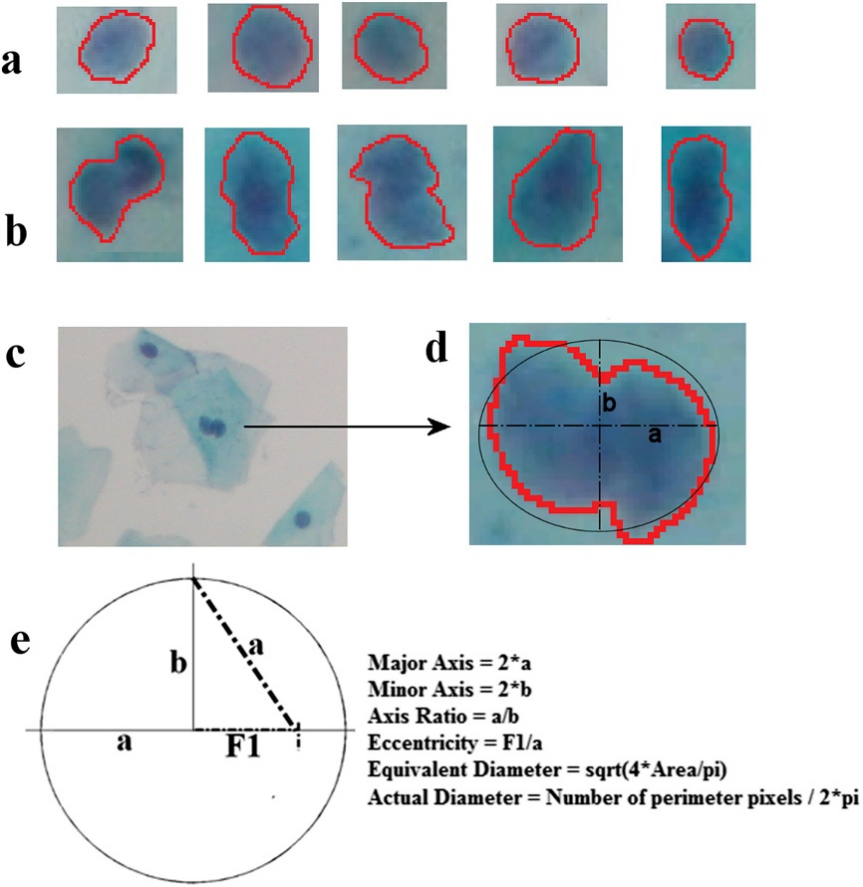
**Extracted features.** Five sample regions are given in **(a)** and **(b)** as an example of both single and overlapped nuclei. Formulation of shape-based features is explained and shown in **(c)**, **(d)**, and **(e)**.

We classified overlapping nuclei in this study by using a fuzzy clustering algorithm. With this approach, extracted nuclei features are clustered into two groups—those with possible overlapping regions and single nuclei features with no overlapping. Hence, the extraction of the most significant features may be crucial for achieving better clustering results. The five features we extracted to identify overlapping nuclei, as listed in Table 1, include three shape-based features supported by two textural features. Variations in the shape and texture between samples with overlapping and single nuclei regions are shown in Figures 4a and 4b, respectively. Also, we introduce the parameters of the shape-based features we utilized in Figures 4c, 4d, and 4e.

**Table 1:** **Extracted features from detected nuclei regions**

1.	Eccentricity*	
2.	Major axis to minor axis ratio*	
3.	Equivalent diameter to actual diameter ratio*	
4.	Number of local minima**	–
5.	Max distance between local minima**	–

^*^a,b and F1 are given in Figure 4e. *P*_n_= number of pixels in perimeter. **Extraction process is given in text.

The shape-based features we utilized depended on the regularity of the nuclei perimeter. We extracted regularity information by evaluating both axes, as shown in Figure 4e. The major and minor axes are basically two lines through the center of an ellipse-shaped object. The difference between the lengths of these two lines is less in the single regions than in the overlapping regions in most of the sample images. Overlapping results in flattened regions, which may be a characteristic appearance. Thus, one of the shape-based features we used was the highly distinctive ratio of these axes for nuclei discrimination [11]. We also extracted the eccentricity of the candidate region in our evaluation, to determine how closely the shape of each object was to an ideal circle, as formulated in Table 1.

The final shape-based feature we extracted was the ratio of the object’s equivalent diameter to the actual diameter, which may significantly change if the boundary of the object is wavy and irregular. Most single nuclei tend to appear as circular smooth objects. High irregularity and/or a wavy regional boundary structure may indicate the presence of overlapping. Formulations of these shape-based features are given in Table 1.

**Figure 5:**
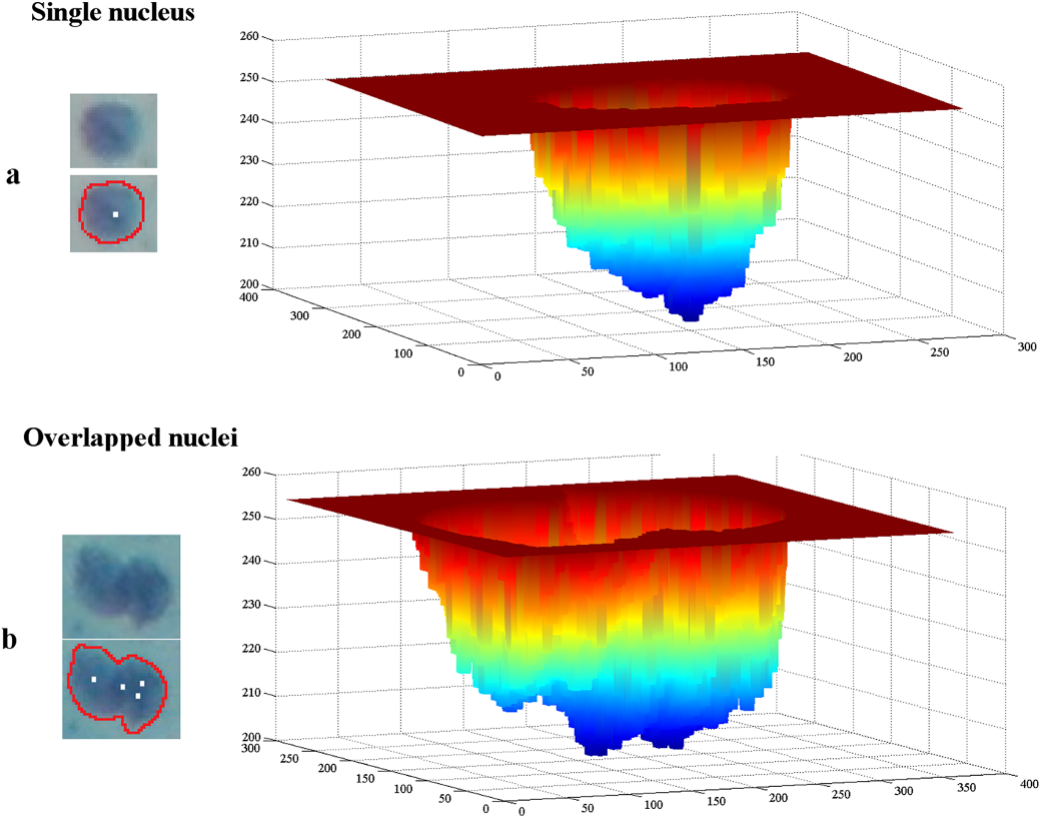
**Visualisation of local minima points.** Local minima points are marked with a white pixel (left handside images) and illustrated with intensity mesh in shades of blue (right handside images) for single and overlapped nuclei regions inside a previously defined boundary. **a)** There is only one local minima. **b)** Overlapping causes multiple intensity valleys inside the region.

In addition to our analyses of shapes, we used two textural features for discrimination purposes. Both of these features were based on the local minima points of delineated nuclei regions. A local minimum point indicates the bottom point of an intensity valley in the image. In contrast with the global minimum, there may be more than one local minimum in the grayscale region. In our study, if a pixel has the lowest grayscale value in a neighborhood set (8-connected), then it is assumed to be a local minimum point [10]. A local minimum for a single nucleus is shown on the intensity mesh in Figure 5a.

Local minima points located inside regions most often indicate higher matter density. We may presume that more than one nucleus inside a candidate region will change the regular density distribution inside the region boundary. The existence of multiple nuclei inside a region causes fluctuations in the bottom points, which then increases the number of local minima [12, 13]. According to this information, increases in the number of local minima points are most likely inside overlapped regions. Increased numbers of local minima points are shown in shades of blue in Figure 5b.

The other textural feature is based on the Euclidean distance between local minima points which gives information about the regularity of the local minima distribution. Using this approach, we extracted the distance between local minima points and assigned the maximum distance as the region’s maximum distance property. The basic approach used to extract the maximum distance of the local minimum points is given in Algorithm 2. 

### D. Classification

**Figure 6:**
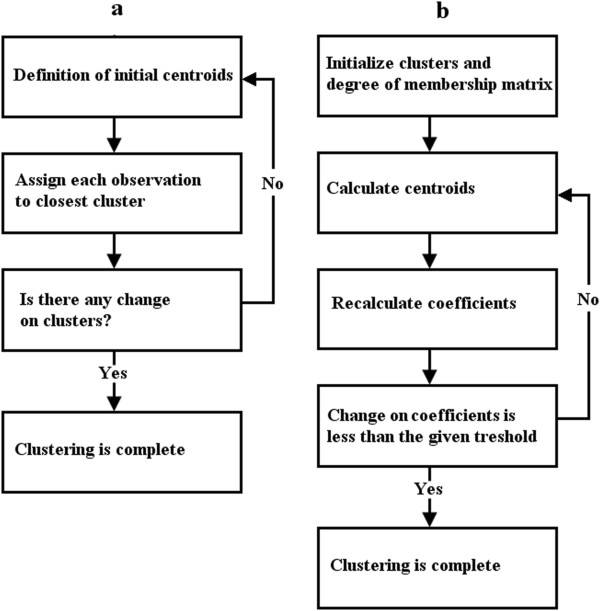
**Computational steps of the examined clustering algorithms.** Block diagrams of examined techniques in the study **(a)** k-means and **(b)** fuzzy c-means.

In this section, we examine fuzzy and non-fuzzy clustering algorithms and compare them with respect to their discrimination of overlapped nuclei. We use the k-means technique for the non-fuzzy approach, which was first introduced by McQueen [14]. The goal of this approach is to partition n-numbered observations into k sets. Since this method is non fuzzy, each of the pattern clusters have one center at any given time. This algorithm updates the centroids with each iteration to minimize the within-cluster sum of the squares, which is defined as 1

where the  term indicates the distance between an observation and the cluster’s centroid. With this approach, the algorithm assigns observations to clusters according to their distance from cluster centers, and updates the centroids of new members. A block diagram of this process is shown in Figure 6a.

The fuzzy c-means (FCM) clustering method, was first introduced by Dunn in 1973 and then improved by Bezdek et al. in 1981. FCM is simply the optimization of the basic c-means objective function using a fuzzy approach. In contrast to k-means clustering, every observation has a degree of association with all sets, according to their distances apart, and observations do not belong to just one cluster. Every point has a set of coefficients [15], each of these coefficients represents a degree of association with one of the clusters, and the centroids of the clusters are the weighted means of the sets.

A point’s degree of belonging to a cluster is inversely proportional to the distance between a cluster centroid and the point. Accordingly, a greater distance means a lower degree of belonging to a set, calculated by: 2

Where, *u*_*ij*_ is the degree of belonging of *x*_*i*_ in the cluster *j*. Also, *c*_*j*_ represents the center of each cluster which is determined by:
3

Clustering is determined by an iterative algorithm. Centers of the clusters and coefficients are updated upon each iteration until the change in coefficients is less than a given threshold. A block diagram of the algorithm we used is shown in Figure 6b. Ultimately, all observations are divided into two main clusters at the end of the iteration process.

## Results

We examined the segmentation capability of this method with respect to 290 nuclei during their development stage. Each of the nuclei were segmented and classified by computer with no human intervention. The experimental algorithm successfully located 87% of the nuclei with 82% sensitivity (true positive rate). We evaluated the segmentation capability of the developed system by comparing the Tanimoto similarity between the empiric and automated segmentation results. The Tanimoto similarity coefficient is widely used for measuring similarities between two binary arrays [16]. It is determined by the ratio of the common to the uncommon bits in the two different arrays. The Tanimoto coefficient provided the rate of similarity between the segmented areas in our study. It may be formulated as: 4

**Figure 7:**
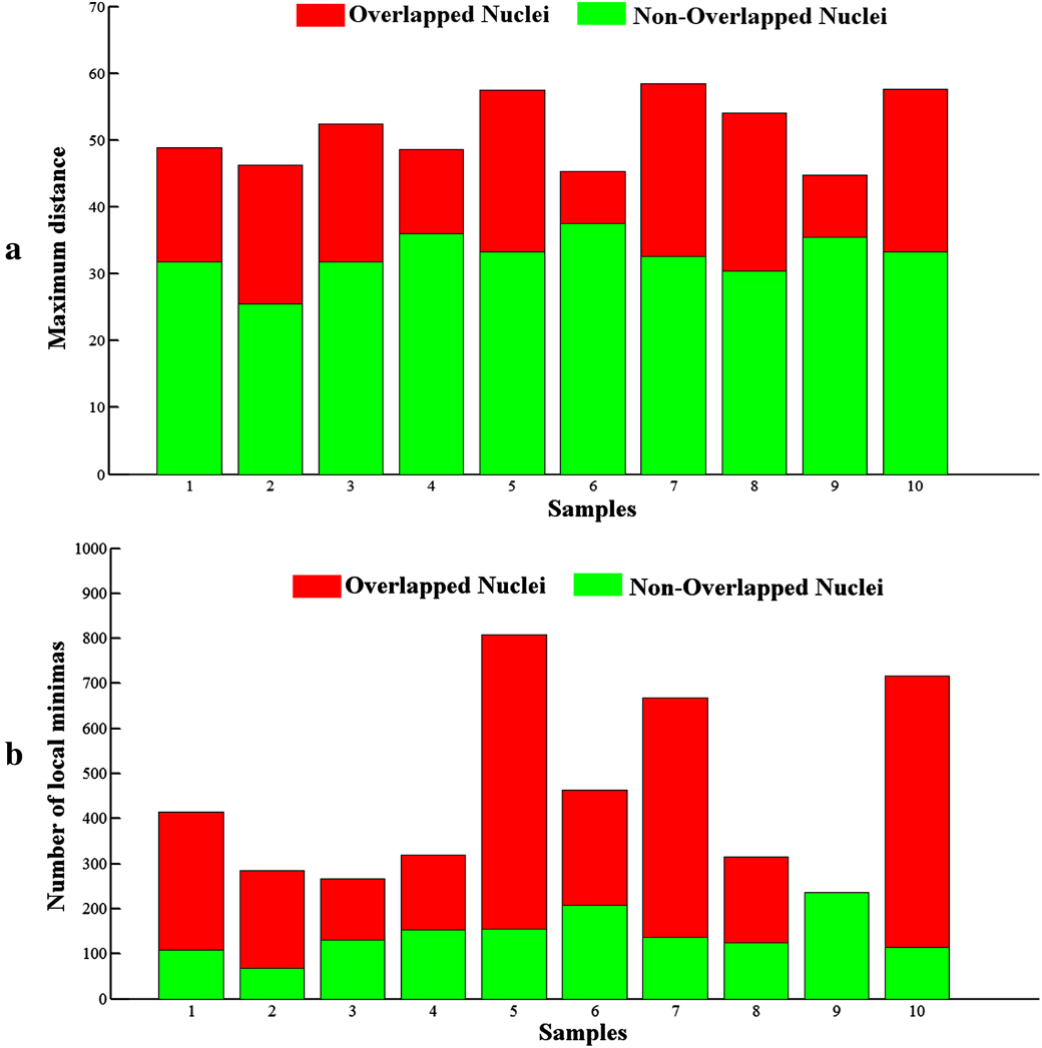
**Variation of local minima points.** Two textural features are observed on twenty cells for two groups as overlapped and non-overlapped. **a)** Difference of distances in cases of overlapping is shown. **b)** Variation of number of local minima inside the region is shown.

In this equation, the Nc term is the number of the common-valued pixels in two images. Na is the number of pixel values which occur only in image a, and Nb is the number of pixel values which occur only in image b. We extracted three binary images from each sample image for comparison. The first holds regions segmented by computer, and the other two are the empirical areas, segmented by two different expert observers. A comparison of the observers’ segmentations were accepted as ground truth. The segmentation success for the 290 nuclei is given in Table 2.In this study, we propose a new combination of features containing two local minima-based features in addition to shape-dependent features. The two textural features extracted for 20 previously segmented nuclei are shown in Figure 7, to highlight the variance of such features in the presence of overlapping.

**Table 2:** **Segmentation success of method for 290 nuclei where tanimoto coefficient utilized as success criteria**

	Observations	Mean	Standard deviation
Proposed method	290	0.732	0.09
Ground truth	290	0.803	0.07

In addition to the information shown in Figure 7, we also compared the significance of the proposed feature set with another set of 5 features. These include: the mean intensity of the region, the minimum intensity value of the region, the area, the convex area, and the frequency with which these regions are used for cervical cell classification [11]. In this test group there are 3 shape- and 2 texture-based features in the proposed feature set as well. We used the independent features significance testing method proposed by Weiss and Indurkhya to compare the significance of both feature sets [17]. Results are given in Table 3.

**Table 3:** **Significance comparison of proposed and test feature sets for 290 Nuclei where independent features significance test utilized for objective comparison**

	Feature 1	Feature 2	Feature 3	Feature 4	Feature 5
Proposed feature set	2.81	4.64	7.74	5.20	4.74
Test feature set	0.77	1.52	3.77	3.91	3.77

For our purposes, malignant and non-malignant nuclei were assumed to be in the same class. We observed variations in the proposed features in the previously segmented and classified nuclei regions from the Herlev data set to justify a feature set as sufficient even if abnormal nuclei were present within the samples. Cluster centroids of features are given in Table 4. for comparison.

**Table 4:** **Cluster Centroids of different classes from independent data sets**

HERLEV data set		
Normal-single		
**Feature**	**Number of nuclei**	**Cluster centroid**
1	140	0.59
2	140	1.36
3	140	2.16
4	140	33.26
5	140	79.04
**Abnormal-single**		
**Feature**	**Number of nuclei**	**Cluster centroid**
1	140	0.62
2	140	1.40
3	140	2.11
4	140	76.04
5	140	160.95
**Test data set**		
**Normal-single**		
**Feature**	**Number of nuclei**	**Cluster centroid**
1	266	0.55
2	266	1.31
3	266	2.79
4	266	16.16
5	266	17.60
**Overlapped nuclei**		
**Feature**	**Number of nuclei**	**Cluster centroid**
1	24	0.86
2	24	2.39
3	24	2.17
4	24	75.27
5	24	48.05

In order to make an objective evaluation of the classification performance of the proposed approach, we compared the empirical and automated classification results with respect to the 3 performance parameters shown below: 5a5b5c

where Tp, Tn, Fp, and Fn are the number of true positive, true negative, false positive, and false negative pixels, respectively. We measured the classification performance of the two clustering approaches using the same experimental environment during the study. These comparison results are given in Table 5, where the “Total Time” column indicates the total elapsed time during the clustering process.

**Figure 8:**
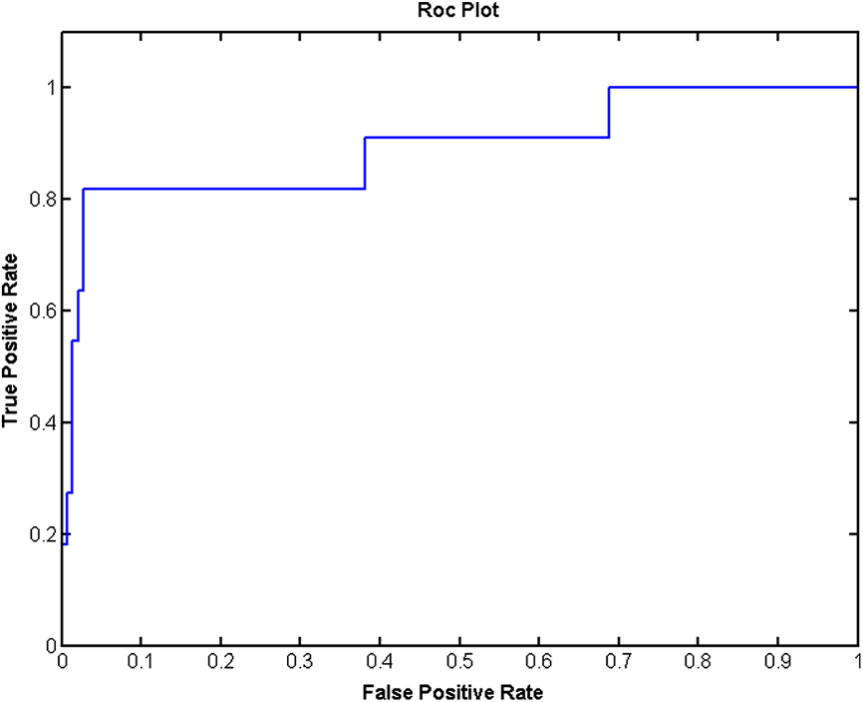
**ROC graphic of proposed method.** Receiver operator characteristics curve is given as a success criteria to the fuzzy c-means algorithm used.

**Figure 9:**
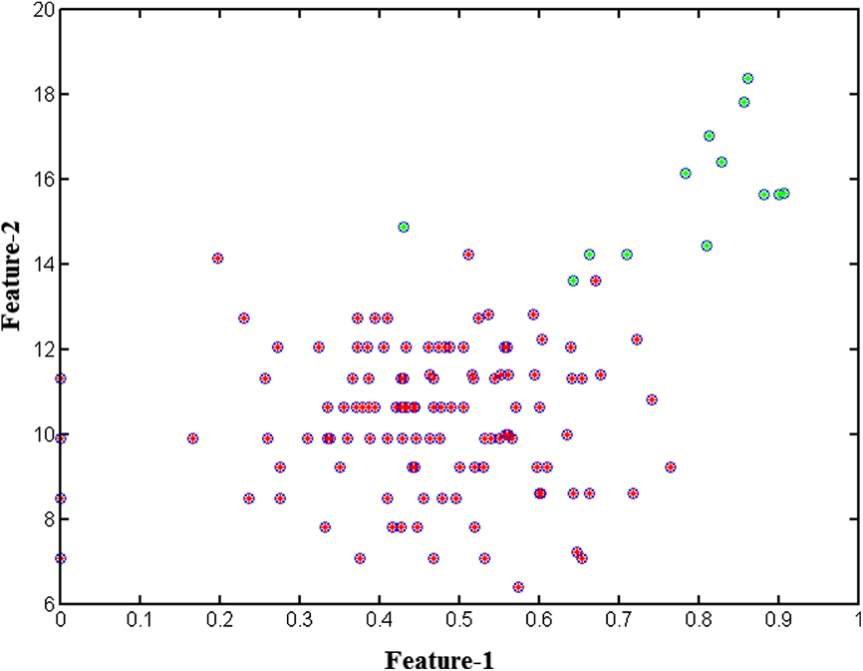
**Clustered features.** Clusters of extracted nuclei on feature space where single and overlapped regions are shown as red and green circles, respectively.

The classification capacity of the proposed approach is also represented with a plot of the graphical receiver operator characteristics (ROC), as shown in Figure 8. The ROC plot uses multiple variables to show that the FCM algorithm may successfully classify overlapped, adjacent nuclei with the proposed features set. The clusters of segmented nuclei at the end of the classification process are shown in Figure 9.

**Figure 10:**
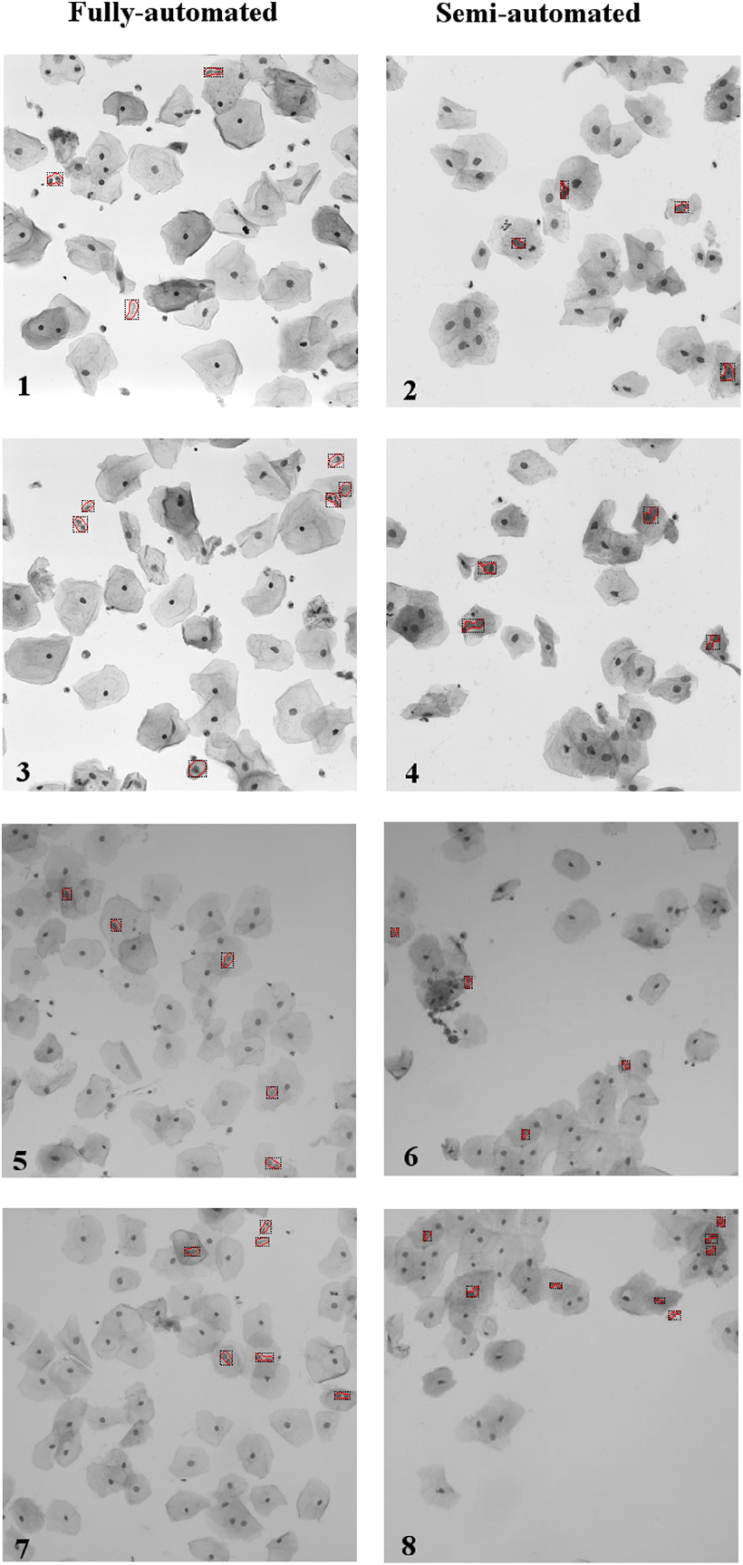
**Visual demonstration of classification success.** Results, generated by proposed methods are presented. Automatically segmented and judged samples are included in fully-automated setup category. Samples, segmented by human eye and analysed with computer are included in semi-automated category. 1,2,3 and 4 numbered samples are from ISBI 14 Challenge data set which are taken from http://cs.adelaide.edu.au/~carneiro/%20isbi14_challenge/dataset.html, 5,6,7,8 numbered samples are picked from our data set. Overlapped regions are indicated by red boundaries and black frames.

Overall success of the system is presented by fully-automated and semi-automated experimental setups in this study. All nuclei are segmented and processed without any human intervention in fully-automated setup. Besides, a semi-automated setup is utilized for observing the affect of segmentation stage on overall decision success. In the semi-automated setup, each of the nuclei within the samples are segmented by human observer, than computer analysed the pre-segmented regions. Outputs of each process with samples from our test set and ISBI data set is given in Figure 10.

**Table 5:** **Classification performance comparison of K-means and fuzzy C-means**

	Classified nuclei	F-Score	Recall	Precision	Time elapsed (ms)
C-means	290	0.791	0.674	0.957	64
K-means	290	0.766	0.666	0.955	145

## Discussion

Overlapping occurs in most of the Pap smear samples in different degrees. Overlapped and adjacent nuclei regions appear mostly as larger, irregular objects in the samples [1]. That excessive growth in size occurs in malignant cases is a matter of a priori knowledge about nuclei in Pap smear samples [2]. Therefore a fully automated classification system for histological abnormalities should be able to differentiate and also separate overlapping/aggregating candidate objects.In this study we proposed a prerequisite approach for a fully automated separation system which involves a pre-classification system for advanced abnormality detection and interregional border extraction of nuclei. Most of the separation studies in the literature do not have any particular detection mechanism for locating and differentiating overlapping/aggregating nuclei. In this study, our goal was to propose an approach that could be used with previously introduced separation methods. Accordingly, we propose a new combination of features for clustering and for detecting overlapping regions even if there are abnormal nuclei inside the regions.

The gradient magnitude of the samples is processed with an edge filter initially, to extract the borders of the nuclei. The actual walls are then filtered from the remaining pixel groups using morphology-based filtering. We evaluated the capability of the proposed basic automated segmentation method by determining the Tanimoto coefficient (also known as Jaccard Index), which is a frequently used similarity measure for evaluating the similarity between two binary images [16, 18]. According to our Tanimoto similiarity criteria results in Table 2, the examined methods are capable of segmenting most of the nuclei regions. There are many studies that prefer similar morphological operations for pre-segmenting or preprocessing cervical cell nuclei [11, 19], and the proposed differentiating mechanism may also be integrated with other automated segmentation methods such as the watershed, active contours, and machine learning-based segmentation approaches [4, 5, 10]. The success of the segmentation stage directly affects the overall classification ability of the proposed approach [12]. Proposed combination of features are also evaluated with semi-automated setup where nuclei were segmented by an observer. Results of semi-automated experiments, presented in Figure 10 showed that, some of the undetected nuclei within the fully-automated test samples are probably an effect of non-adaptive nature of preferred segmentation methods. It should be noted that, our approach may be highly compatible with semi-automated systems or a better adaptive segmentation mechanisms. An adaptive segmentation approach, perhaps based on a non-linear decision mechanism, could be adapted in future work to increase the detection capability.

We combine size and textural features in this study to achieve optimum results. We also evaluated the significance of the proposed feature combination by comparing it with an alternative feature set which is formed by frequently used features for classification of nuclei. Both feature sets were then compared with samples from the test data set for 290 nuclei. Results of this comparison given in Table 3 showed that the proposed feature set achieves a higher level of significance for nuclei overlapping. In previous studies, similar feature sets were used for segmentation and separation of overlapped nuclei [5, 11, 12]. Also, the experiments in previous studies showed that conducting a clustering analysis on size-dependent features only may not be sufficient for recognition of overlapping [3]. So, there are also many studies that have combined textural and shape-based features [5, 11, 12]. However, the combination of features we introduce in this study is unique for use in the discrimination of overlapping. It should be noted that most studies did not have any particular mechanism for classifying overlapped regions before the segmentation process. Usually, morphological operations or alternative preprocessing stages were carried out prior to any further analyses [1, 3]. Methods, introduced in the study should be seen as a supporting approach to potentially increase the separation capabilities of existing overlapping nuclei segmentation methods.

In the present work, we classified the extracted nuclei features from nuclei using clustering-based methods. Since there is no need for a training set or stage with data clustering approaches, this system may be promising for the varying conditions of different samples. We also examined and compared two well-known fuzzy (FCM) and non-fuzzy (k-means) clustering approaches [14, 15]. According to Table 5, both of these methods are capable of discriminating overlapping. However the fuzzy c-means is faster and has a higher f-score, so it is computationally more effective and a better choice for our work. In addition, some consideration should be given to the idea that an optimizing fuzzy clustering approach may increase the classification capability [20].

The proposed features were also examined with samples from the Herlev data set, a well-known data set frequently used for performance testing benchmark data [21]. Samples from the HDS were pre-classified and segmented. These samples are preferred for determining the centroids of clusters, since the data include both malignant and normal samples. We expected that the developed system would cluster overlapped and non-overlapped nuclei even in data containing abnormal cells data.

Table 4 presents that, both of the textural features tend to increase due to expanded area of nuclei in abnormal cases. However, it should be noted that most of the nuclei preserves it’s circular or ellipsoid structure in abnormal cases which is also indicated in the table. All shape based feature centroids are closer to normal single nuclei centroids in abnormal class in Table 4. Moreover textural features tend to change more significantly in overlapped class. In the presented data centroids of malignant and normal cell features are closer in value, which may indicate that abnormal and normal nuclei are most likely being classified in the same cluster.

Previous studies show that, features extracted from both cytoplasmic region and nuclei are essential for detection of abnormality in an automated Pap smear screening system [2, 5, 7, 22]. We proposed methods for discrimination of overlapped nuclei which should be suggested as an elimination mechanism before feature extraction for abnormality detection [3]. As a result of refined samples from overlapped regions, classification abilities of automated systems are expected to be improved. It should be noted that eliminated overlapped regions can be separated in further stages for searching abnormality inside the region.

## Conclusions

The developed and proposed methods in this study may be considered as a supporting approach for studies of the segmentation of interregional borders of nuclei where overlapping occurs. Our method does not depend on a certain quantity of nuclei inside the region. In fact, greater numbers of nuclei inside a region may be an advantage for classifying local minima-based features. In a practical sense, the main contribution of our method is as a pre-classification approach which includes specialized features for effective discrimination despite the varying overlapping conditions. We hope this study may serve as a new basis for further studies in automated Pap smear screening.
